# A Risk Factor for Attention Deficit Hyperactivity Disorder Induces Marked Long-Term Anatomical Changes at GABAergic-Dopaminergic Synapses in the Rat Ventral Tegmental Area

**DOI:** 10.3390/ijms252312970

**Published:** 2024-12-02

**Authors:** Steve Seo, Louise C. Parr-Brownlie, Hollie E. Wicky, David K. Bilkey, Stephanie M. Hughes, Dorothy E. Oorschot

**Affiliations:** 1 Department of Anatomy, School of Biomedical Sciences, University of Otago, Dunedin 9054, New Zealand; sseo73@gatech.edu (S.S.); louise.parr-brownlie@otago.ac.nz (L.C.P.-B.); 2 Brain Health Research Centre, University of Otago, Dunedin 9054, New Zealand; hollie.wicky@gmail.com (H.E.W.); david.bilkey@otago.ac.nz (D.K.B.); stephanie.hughes@otago.ac.nz (S.M.H.); 3 Brain Research, Dunedin 9054, New Zealand; 4 Department of Biochemistry, School of Biomedical Sciences, University of Otago, Dunedin 9054, New Zealand; 5 Department of Psychology, University of Otago, Dunedin 9054, New Zealand

**Keywords:** rostromedial tegmental GABAergic neurons, dopaminergic posterior ventral tegmental neurons, immunonanogold, serial section transmission electron microscopy, stereology, schizophrenia

## Abstract

Attention deficit hyperactivity disorder (ADHD) is a common neurodevelopmental disorder. However, the core biology of the disorder that leads to the hypofunctioning of the cerebral dopaminergic network requires further elucidation. We investigated midbrain synaptic changes in male rats exposed to repeated hypoxia during the equivalent of extreme prematurity, which is a new animal model of the hyperactive/impulsive presentation of ADHD. We used a novel combination of a lentiviral vector, peroxidase-immunonanogold double-labelling, three-dimensional serial section transmission electron microscopy and stereological techniques to investigate the synapses formed between GABAergic axons of the rostromedial tegmental nucleus (RMTg) and dopaminergic neurons of the posterior ventral tegmental area (pVTA). This is a key site that sends extensive dopaminergic projections to the forebrain. We also compared the results to our previous study on a schizophrenia risk factor that produces cerebral hyperdopaminergia. In total, 117 reconstructed synapses were compared. Repeated hypoxic rats had a significantly thicker (22%) and longer (18%) postsynaptic density at RMTg GABAergic-pVTA dopaminergic synapses compared to their controls. These results were opposite to those previously observed in rats exposed to a schizophrenia risk factor. These findings for repeated hypoxic rats suggest that the enhanced inhibition of pVTA dopaminergic neurons may contribute to hypodopaminergia in ADHD motor hyperactivity. Synaptic triads, a key component of pVTA circuitry, were not detected in repeated hypoxic rats, indicating a marked deficit. The current knowledge may guide development in males of novel, site-specific ADHD drugs, which is necessary due to the rising prevalence of ADHD, the chronic nature of ADHD symptoms and the limitations of the currently available medications.

## 1. Introduction

Attention deficit hyperactivity disorder (ADHD) is a common neurodevelopmental disorder in children, affecting ~5%, and continuing into adulthood for ~2.5%, of the population [1,2]. ADHD is a debilitating and complex chronic condition with three different presentations (i.e., subtypes): hyperactive–impulsive, inattentive, or combined [3]. The underlying neuropathology is yet to be fully elucidated for each presentation [4].

While various theories of ADHD have been proposed, the dopamine deficit theory continues to be the most prominent [5,6,7]. This theory highlights the hypofunctioning of the cerebral dopaminergic network (i.e., hypodopaminergia) as the neurobiological basis of ADHD symptoms. Consistent with this theory is the mechanism of action of the most effective and widely used medications for the treatment of ADHD (e.g., methylphenidate or amphetamines). These medications are dopamine reuptake inhibitors, thereby increasing the presence of dopamine in the synaptic cleft and extraneuronal space and prolonging its action in the brain [2]. However, these currently available medications are associated with non-response in around 30% of patients, adverse effects (e.g., insomnia, appetite suppression, low adherence), a multitude of drug-to-drug interactions and contraindications and the potential for abuse [2,5,6,8,9,10,11,12,13,14]. Abuse can involve addiction and overuse, with both involving excessive concentrations of dopamine at synapses of the reward pathway between midbrain dopaminergic neurons and the striatum [5]. Hence, it is critical to understand the core biology (dopamine dysregulation) of ADHD in order to develop targeted therapies that could overcome the limitations of currently available medications.

Dopaminergic neurons in the ventral tegmental area (VTA) of the midbrain send extensive projections to the striatum and cerebral cortex [15]. The posterior VTA (pVTA), in particular, contains a high density of dopaminergic neurons [16]. The VTA dopaminergic neurons are under GABAergic inhibitory modulation from five brain regions [17]. Of these, the input from the rostromedial tegmental nucleus (RMTg) acts as a major inhibitory brake on their bursting activity [18,19]. Since dysfunction in the VTA dopamine pathway to the forebrain is implicated in ADHD [20], ADHD is a chronic condition [21] and long-term changes in nervous system function are associated and/or maintained by changes in synaptic structure [22], we hypothesized that there are structural synaptic alterations in the RMTg GABAergic-pVTA dopaminergic circuit that could contribute to the dopamine dysregulation in ADHD.

**Figure 1:**
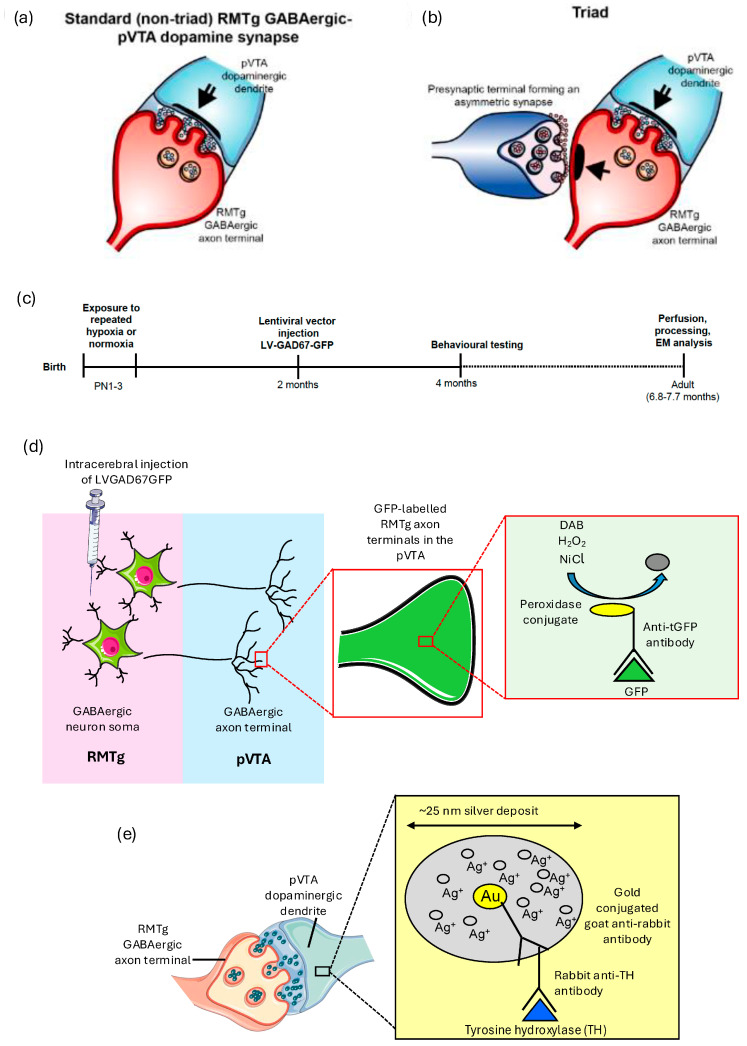
Schematic diagrams of the observed synapses within the pVTA that are referred to as non-triads or triads, a timeline of the experimental procedures and the double immunohistochemical methods. Schematic representation of observed non-triadic (**a**) and triadic (**b**) synapses within the pVTA, based on the electron microscopic images (see later Figures). The single-shafted arrow indicates an asymmetric synapse formed between a presumed glutamatergic axon terminal and a RMTg GABAergic axon terminal. The double-shafted arrow indicates a symmetric synapse formed between a RMTg GABAergic axon terminal and a pVTA dopaminergic dendrite. (**c**) Experimental timeline for the current study. From 4 months-of-age, the rats were behaviourally tested for hyperactivity on a staircase test and then on a fixed interval-extinction test in operant chambers. (**d**,**e**) Schematic diagram showing the methods for immunolabelling, within the pVTA, of the RMTg neuronal profiles using 3,3′-diaminobenzidine tetrahydrochloride (DAB) (**d**) and the dopaminergic profiles using silver-enhanced nanogold (**e**) as the end-label with an expected minimum size of ~25 nm. PN, postnatal day. Please see the main text for the definition of the other abbreviations. (**a**,**b**,**d**,**e**) are from Seo et al. [31], with copyright approval.

Analyses of this synaptic circuitry can only be undertaken in animals [23]. Hence, we investigated the structure of synapses formed between the axons of the RMTg GABAergic neurons and pVTA dopaminergic neurons (Figure 1a) in a repeated hypoxic rat model of ADHD-like hyperactivity and impulsivity [24,25,26]. In this model, adult male rats exposed to repeated hypoxia during the equivalent of human extreme prematurity exhibit ADHD-like hyperactivity and impulsivity, but not inattention [24,25]. The observed behaviour closely resembles the hyperactive–impulsive presentation of human ADHD [7,24,27]. ADHD is the most common neurobehavioral disorder in children and adolescents born extremely prematurely [28,29,30]. We also investigated the putative glutamatergic inputs onto RMTg GABAergic axon terminals that contribute to triadic synapses within the pVTA (Figure 1b). These axoaxodendritic triads were recently discovered in 3-month-old and 14-month-old control Sprague Dawley rats and in rats exposed to maternal immune activation (MIA), a schizophrenia risk factor [31]. These triads are thought to enhance RMTg GABAergic transmission to the pVTA dopaminergic neurons.

We also hypothesized that any synaptic changes in this region in the ADHD-like hyperactive and impulsive rats may be opposite to those observed in our MIA study [31], as schizophrenia is considered to involve a hyperdopaminergic condition in mesolimbic circuitry [32].

**Figure 2:**
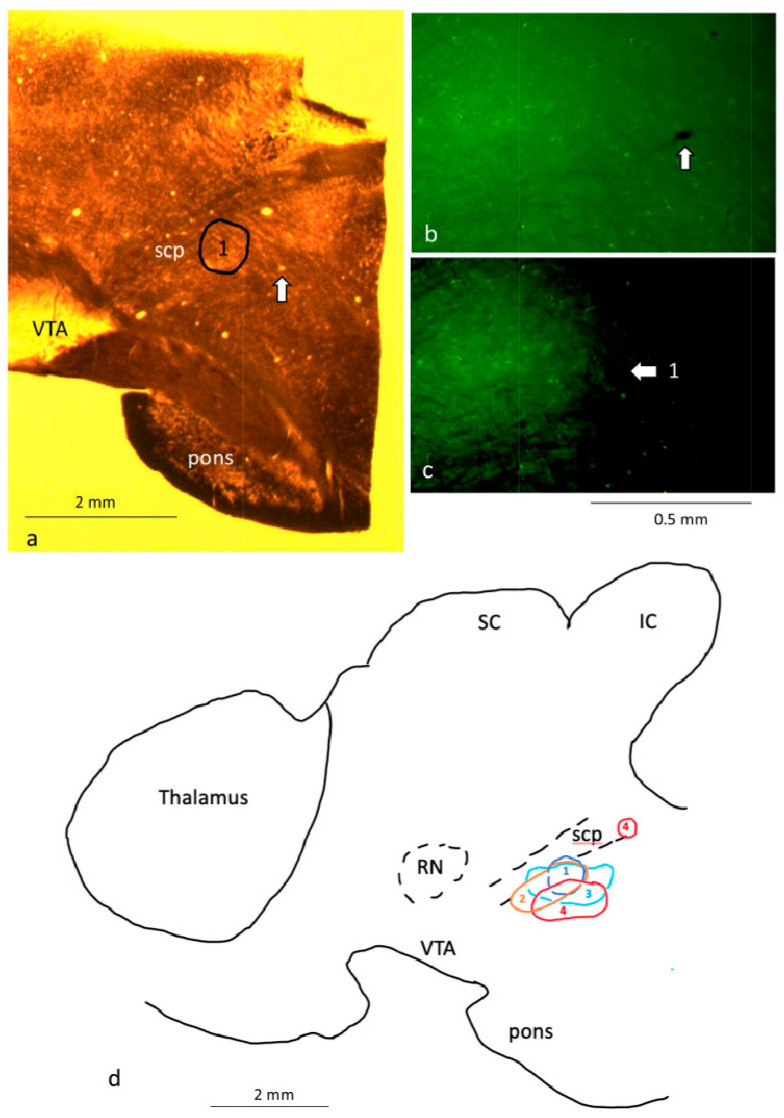
Identification of the rostromedial tegmental nucleus (RMTg) and posterior ventral tegmental area (VTA) at the light-microscopic level. (**a**) Brightfield microscopic image of a brain section, from a 7-month-old repeated hypoxic rat, that has been osmicated and flat embedded in resin after immunohistochemical processing. The location of labelled fluorescent neurons in the RMTg is indicated at (1). The arrow indicates a blood vessel that was used as one of the landmarks to identify the labelled neurons in the fluorescent light microscopic image in (**b**). (**c**) is an image of the same section and region as in (**b**), with the contrast enhanced in (**c**) to identify the main location of the labelled neurons. Note that the image in (**b**,**c**) of a wet-mounted section was taken at a slightly different counter-clockwise angle to the same section photographed in (**a**) after flat embedding in resin. The labelled neurons indicated at 1 in (**c**) were positioned at location 1 in (**a**,**d**). (**d**) Schematic diagram of a sagittal section through the medial rat brain indicating the location of the fluorescent neurons in the RMTg of the four rats analyzed ultrastructurally in this study. The respective location in two repeated hypoxic rats is indicated in dark blue (1) and light blue (3). The respective location in two repeated normoxic rats is indicated in orange (2) and red (4, with two locations observed for rat 4). Rats 1 and 2 were littermates, and Rats 3 and 4 were littermates in a different litter. For a comparison, see Figure 1 in Zhou (2021, [19]). IC, inferior colliculus; RN, red nucleus; scp, superior cerebellar peduncle; SC, superior colliculus; VTA, ventral tegmental area. Scale bars: a,d, 2 mm; b,c, 0.5 mm.

**Figure 3:**
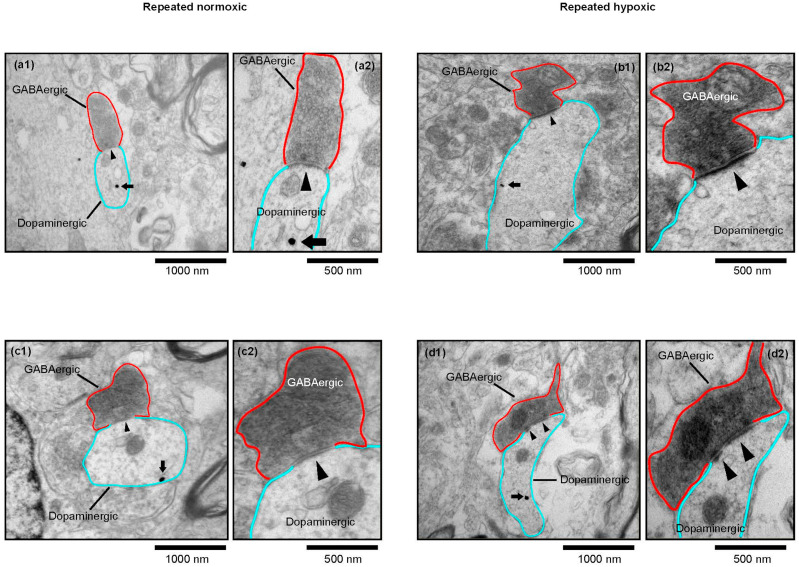
Representative electron micrographs of RMTg GABAergic-pVTA dopaminergic synapses in repeated normoxic and repeated hypoxic rats. (**a1**–**d1**) A representative electron microscopic image of a non-triadic synapse from each repeated normoxic (**a1**,**c1**) or repeated hypoxic (**b1**,**d1**) rat. Each image shows a RMTg GABAergic axon terminal (dark peroxidase-labelled profile outlined in red) making a symmetric synapse (black arrowhead) onto a pVTA dopaminergic dendrite (outlined in turquoise and containing an immunogold particle indicated by a black arrow). Note that immunogold particles were more extensively present in each postsynaptic dendrite than is evident, and could be displayed, in these high magnification images that illustrate synapses. A more extensive presence of immunogold particles is evident in serial sections through the same postsynaptic dendrite (Figure 4a1,a4) or at a similar or lower magnification in other postsynaptic dendrites (Figure 4b1,b2,c1–c3). In Figure 3, synapses in (**a1**–**d1**) are shown more clearly in (**a2**–**d2**), which are images of the same sections taken at a higher magnification. In (**a2**–**d2**), the postsynaptic density (PSD) of the GABAergic synapse appears longer and thicker in the repeated hypoxic rats (**b2**,**d2**) compared to the control repeated normoxic rats (**a2**,**c2**).

To conduct comprehensive analyses of identified synapses at the ultrastructural level at ~7 months-of-age (Figure 1c), we used a novel combination of methods that were established and validated in Seo et al. [31]. Specifically, these were (1) lentiviral (LV) technology, to selectively label RMTg GABAergic synaptic inputs (Figure 1d), (2) peroxidase-immunonanogold double-labelling, to identify GABAergic presynaptic and dopaminergic postsynaptic components (Figure 1d,e), (3) three-dimensional serial section transmission electron microscopy, to unambiguously identify synapses, and (4) stereology to quantify the identified synapses.

## 2. Results

To achieve the comprehensive analyses of identified synapses at the ultrastructural level, two male repeated hypoxic ADHD-like rats and two control male repeated normoxic rats, one of each from two different litters, were investigated into adulthood (i.e., at 7 months-of-age, Figure 1c, see further details below). In total, 33 identified synapses were reconstructed in three dimensions and stereologically analyzed. The synaptic results were also compared with 84 synapses that were investigated using the same three-dimensional methods in Seo et al. [31]. Males were investigated because they are at greater risk of developing ADHD-like behaviour after extreme prematurity [33]. In a similar three-dimensional ultrastructural study on identified synapses, Pan et al. [34] investigated two animals (mice) per group.

The statistical analyses of our anatomical data were based on the number of synapses reconstructed in each group rather than the total number of animals used in this study. This statistical approach is routinely used in ultrastructural studies on three-dimensionally reconstructed synapses, since the method requires extensive serial sections [31,34,35,36,37]. For example, in the current study, for each rat, 28–65 consecutive serial ultrathin sections containing the pVTA were required to complete the three-dimensional quantitative analyses on doubly immunostained identified synapses.

Sections containing the RMTg were first examined using an Olympus AX70 fluorescent microscope to confirm GFP expression in the region (Figure 2). Sections containing the pVTA were then processed further for immunohistochemistry and electron microscopy (EM). Further details are provided in the Materials and Methods (Section 4).

### 2.1. RMTg GABAergic Axons That Synapsed with Dopaminergic Dendrites in the pVTA Contributed to Synaptic Triads in Control Repeated Normoxic Rats at ~7 Months-of-Age

**Figure 4:**
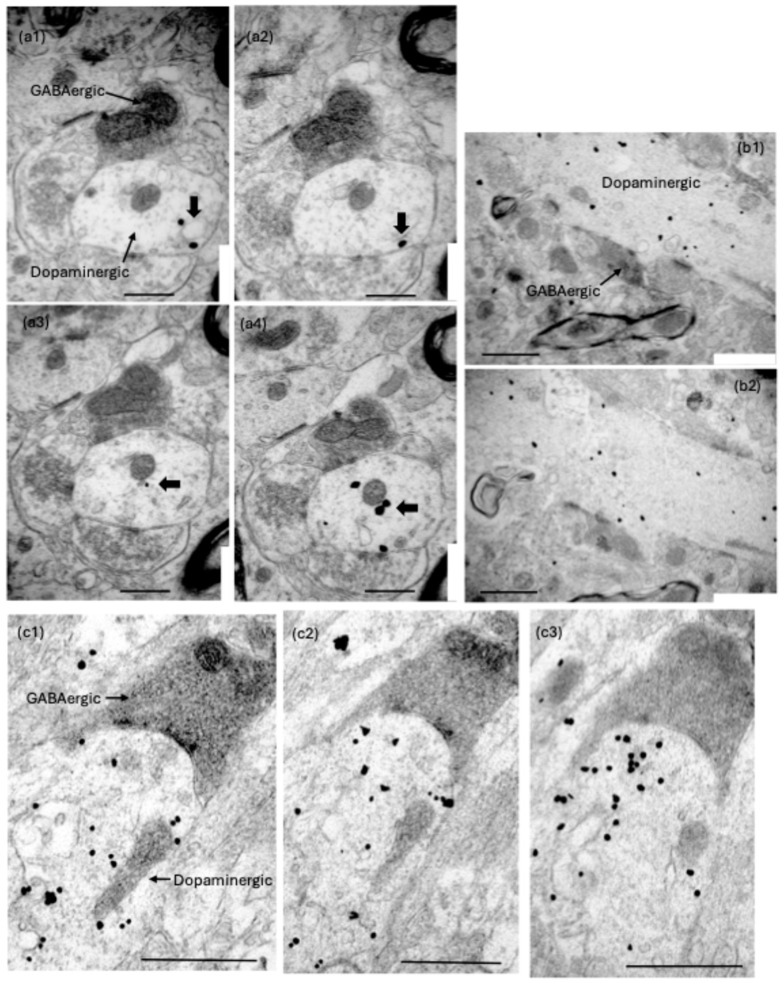
Evidence of a more extensive presence of immunogold particles in serial sections through dopaminergic postsynaptic dendrites in the rat pVTA that are innervated by an RMTg GABAergic presynaptic terminal. (**a1**–**a4**) Serial sections through the postsynaptic dendrite of the synapse (**c1**,**c2**) in Figure 3, which is from a repeated normoxic rat. In these four serial sections (Sections 27–30), note the consistent evidence of one-to-five tyrosine hydroxylase-positive immunogold particles (short black arrows) that are specific to the postsynaptic dendrite and are not evident in the adjacent tissue. Note also the consistent immunostaining of the presynaptic peroxidase-positive RMTg GABAergic terminal and its synapse. All these features are also evident for another measured synapse in this study (**b1**,**b2**) from a repeated hypoxic rat in two serial sections (Sections 27–28). They are also evident in another measured synapse (**c1**–**c3**) from a repeated normoxic rat in three serial sections (Sections 44–46). Scale bars: (**a1**–**a4**), 500 nm; (**b1**,**b2**), 1000 nm; (**c1**), 890 nm, (**c2**,**c3**), 825 nm.

**Figure 5:**
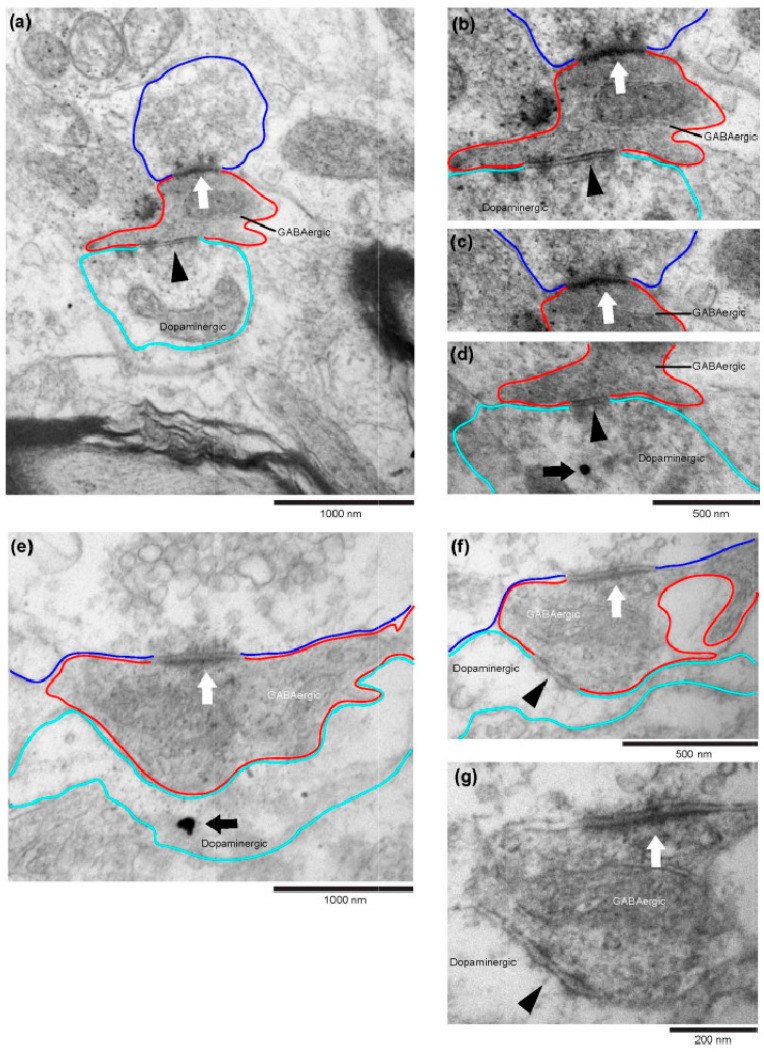
Evidence of synaptic triads within the pVTA of a ~7-month-old control repeated normoxic rat. Two different synaptic triads are illustrated, one in (**a**–**d**) and another in (**e**–**g**). The electron microscopic images show an unlabelled, presumably glutamatergic, axon profile (outlined in dark blue in each synaptic triad in (**a**,**e**)) that makes an asymmetric synapse (white arrow) onto a copGFP-positive dark peroxidase-labelled RMTg GABAergic axon terminal (outlined in red). This GABAergic axon terminal, in turn, makes a symmetric synapse (black arrowhead in (**a**,**b**,**d**,**f**,**g**)) onto another profile (outlined in turquoise). This was confirmed in an adjacent serial section (**d**) of the first synaptic triad as a TH-positive, immunogold-labelled (black arrow) dopaminergic dendrite. A TH-positive, immunogold-labelled (black arrow) dopaminergic dendrite is indicated in (**e**) for the second synaptic triad. For the first synaptic triad, the symmetric synapse in (**a**) between the RMTg GABAergic axon terminal and pVTA dopaminergic dendrite is shown more clearly in (**b**), which is a higher magnification image of the same section in (**a**). The asymmetric synapse in (**a**), between the presumed glutamatergic synapse and the RMTg GABAergic axon terminal, is more clearly identifiable in (**c**), which is a higher magnification of the same section shown in (**a**,**b**). The image in (**c**) has been slightly tilted using a goniometer function in the electron microscope. For the second synaptic triad, the asymmetric synapse (white arrow) and the symmetric synapse (black arrowhead) are shown more clearly in (**g**), which is a higher magnification image of the same section in (**f**).

In control repeated normoxic rats, peroxidase-labelled RMTg GABAergic axon terminals synapsed onto immunogold-labelled pVTA dopaminergic dendrites (Figure 1a and Figure 3a1,a2,c1,c2). Immunogold-labelled dendrites were evident across serial sections (Figure 4), confirming their identity. A subset of these GABAergic axon terminals received an asymmetric, presumably excitatory glutamatergic [38], synaptic input (Figure 1b and Figure 5). These asymmetric synapses had densely packed synaptic vesicles (Figure 5b,c), consistent with glutamatergic inputs previously reported in the VTA [39]. Hence, we confirmed previous findings [31] of the presence of axoaxodendritic synaptic triads in control rats and extended these findings to include animals of ~7 months-of-age. Specifically, the observed synaptic triads have been previously reported in the pVTA of 3-month-old and 14-month-old rats [31]. 

### 2.2. Synaptic Triads Were Not Present in Repeated Hypoxic Rats

In contrast to the control repeated normoxic rats, repeated hypoxic rats displayed no evidence of an asymmetric synaptic input onto RMTg GABAergic axon terminals that synapsed onto pVTA dopaminergic synapses. Hence, all RMTg GABAergic-pVTA dopaminergic synapses in the repeated hypoxic rats were non-triadic, standard synapses (Figure 1a and Figure 3b1,b2,d1,d2). This is a unique and important finding since synaptic triads occur frequently in the pVTA at labelled RMTg inputs. In Seo et al. [31], we found them within a few synapses when analyzed in three dimensions.

### 2.3. RMTg GABAergic Synapses on pVTA Dopaminergic Dendrites Were Significantly Thicker and Longer in Repeated Hypoxic Rats Compared with Repeated Normoxic Rats

**Figure 6:**
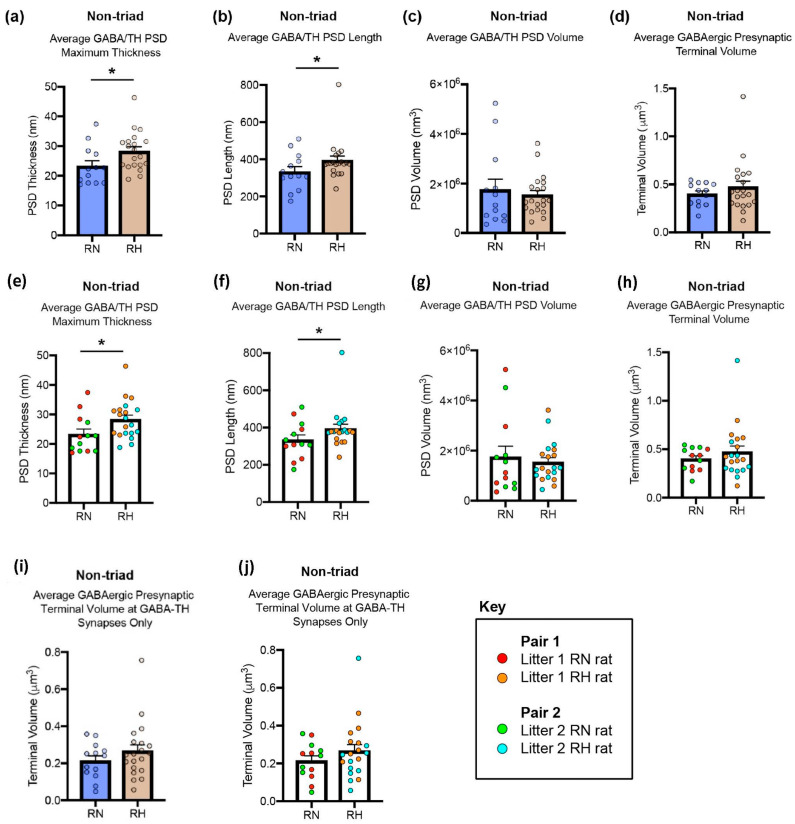
Quantitative comparison of the ultrastructure of RMTg GABAergic-pVTA dopaminergic non-triadic, standard synapses in control repeated normoxic rats and repeated hypoxic rats (**a**–**d**,**i**). Variation within each rat for the synaptic data is also illustrated (**e**–**h**,**j**). Repeated hypoxic rats had a statistically significant thicker (**a**) and longer (**b**) PSD (Mann–Whitney *U* test, two-tailed, *U* = 66, *p* = 0.0172, and Mann–Whitney *U* test, one-tailed, *U* = 81, *p* = 0.0368, respectively) compared to the repeated normoxic controls. There was no statistically significant difference in PSD volume (**c**, *U* = 111, *p* = 0.5009) between the two groups. There was also no difference between the two groups when the RMTg GABAergic presynaptic volume was measured across the full span of the terminals ((**d**), *U* = 123, *p* = 0.8134) or only in the serial sections that contained the identified GABAergic–dopaminergic synapses ((**i**), *U* = 108, *p* = 0.4337). Graphical illustrations of the intra-group variability for the ultrastructural quantitative measures that showed a statistically significant difference between the repeated normoxic and repeated hypoxic rats (**a**,**b**) and those that did not (**c**,**d**,**i**) are provided in (**e**–**h**,**j**). These graphs illustrate that the data are representative across animals of their respective groups and that no animal carried the findings. Specifically, when the highest four values in each group were examined, for all but one measure, the highest four values were a mix of either rats within each repeated hypoxic or repeated normoxic group. Even for the one measure that did not have “mixed values” ((**f**): average GABA/TH PSD length), it is evident that the higher values for the repeated hypoxic and repeated normoxic rats belong to paired littermates (see the Key), whereas the lower values belong to another set of paired littermates (i.e., the increase for repeated hypoxia versus repeated normoxia is consistent across paired repeated hypoxic/repeated normoxic rats). Hence, these scatterplots indicate that data from one rat (in either group) are not skewing the findings and generating the observed statistical significance. Significance was assessed by a Mann–Whitney *U* test, based on normality testing of the data. The data are expressed as the mean ± S.E.M. * *p* < 0.05. RN, repeated normoxic; RH, repeated hypoxic. These data are illustrated schematically in a later Figure.

Since triadic synapses were only observed in the control repeated normoxic rats, quantitative comparison of the synaptic circuitry between repeated normoxic and repeated hypoxic rats was only possible for non-triadic synapses. Statistical analyses of the quantitative data were based on 13–20 identified synaptic non-triads per group (i.e., 6–10 synaptic non-triads per rat). Compared to the repeated normoxic controls, the repeated hypoxic rats had a statistically significant thicker (22%) and longer (18%) PSD at RMTg GABAergic-pVTA dopaminergic non-triadic synapses (Figure 6a,b). The specific measurement compared was the maximum thickness, and the maximum length, respectively, across the entire span of the identified synapses. These anatomical alterations could underlie the enhanced GABAergic inhibition of the pVTA dopaminergic neurons, leading to decreased dopamine release. In the same synapses, there was no difference in the PSD volume (Figure 6c, see also the Discussion, Section 3) and the RMTg GABAergic presynaptic terminal volume (Figure 6d,i). Analysis of the individual variation per rat for the foregoing data is provided in Figure 6e–h,j. These analyses indicate that the data are representative across animals of their respective groups and that no one animal carried the findings.

**Figure 7:**
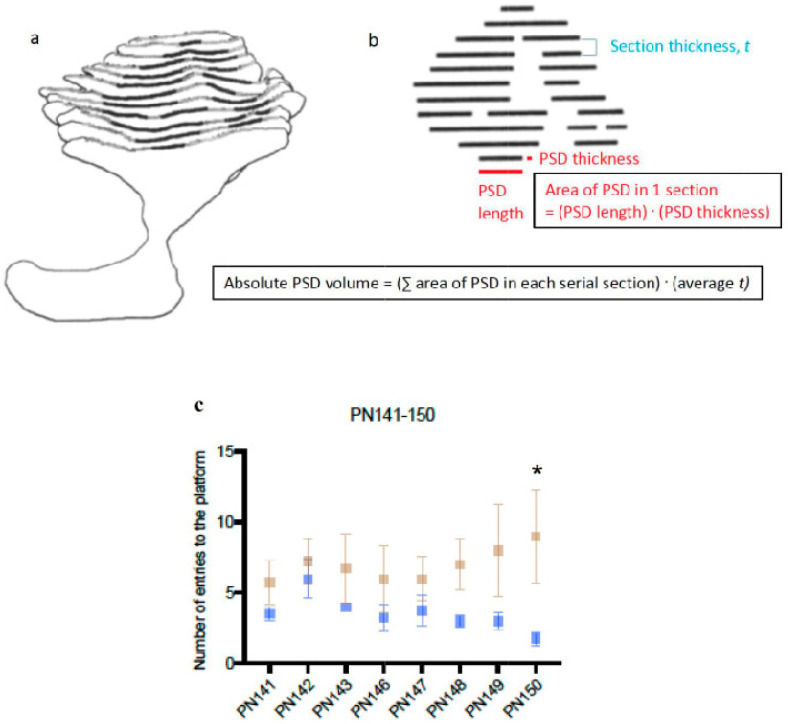
Schematic diagrams of a postsynaptic density (PSD, solid black lines) in consecutive serial sections to illustrate the stereological methods used, and behavioural results. The entire PSD belongs to one presynaptic terminal. In (**a**), the perforations (gaps) within the entire PSD are illustrated. In (**b**), there is an en-face view (i.e., view from above) of the entire PSD that is simplified to a line diagram. This view illustrates the principle of the Cavalieri stereological method that was used to measure the absolute (i.e., total) volume of a PSD. Note that the real anatomy as shown in (**a**) was used to complete the actual measurements. (**a**,**b**) These are sourced from Jones and Calverley [40] for illustrative purposes only, with copyright approval. (**c**) Comparison of activity of repeated normoxic rats (blue squares) and repeated hypoxic rats (brown squares) during staircase testing. From PN141 to PN150, the two repeated hypoxic rats made a significantly higher number of entries onto the platform, per daily 5 min testing session, than the two repeated normoxic rats (two-way repeated measures ANOVA of the interaction between repeated normoxia versus repeated hypoxia and the performance on the right and left side of the staircase test, F_7,42_ = 2.725, *p* = 0.0200). Thus, across time, the repeated hypoxic rats were significantly more hyperactive in traversing to and fro between the platform and the entry chamber of the staircase test. Due to the time-consuming nature of stereological electron microscopic studies on synapses, the behavioural data from the same animals was statistically analyzed using the performance on the right and left side of the staircase test as independent variables for each animal. Post hoc Holm’s-Sidak multiple comparisons tests, on the specific data for each day, revealed a significant difference at Week 3, Day 5 (PN150, *p* = 0.041). The two-way repeated measures ANOVA showed no significant difference for time alone or treatment alone. The data are expressed as the mean ± S.E.M. * *p* < 0.05.

Qualitatively, we observed that there may be more synapses with complex perforations (Figure 7a,b) in the PSD for the repeated hypoxic rats. On quantitation, the number of synapses with simple perforations was 11 for the repeated hypoxic rats and 9 for the repeated normoxic rats. The repeated hypoxic rats had a higher incidence of complex perforations in the PSD of a presynaptic terminal (*n* = 1 or 4 synapses per rat, total of 5 synapses, 14–44%) compared to 0% (*n* = 0) for the repeated normoxic rats, although this difference was not statistically significant (one-tailed Fisher’s exact test, *p* = 0.061).

### 2.4. The Foregoing Quantitative Data Were Corrected for Processing-Induced Shrinkage

The average area shrinkage was 0.9859 (1.41%) and 0.9820 (1.80%) for repeated normoxic rats and 0.9762 (2.38%) and 0.9608 (3.92%) for repeated hypoxic rats. The average linear shrinkage was 0.9929 (0.71%) and 0.9910 (0.90%) for repeated normoxic rats and 0.9880 (1.20%) and 0.9802 (1.98%) for repeated hypoxic rats. This difference could be attributed to one or more of glial swelling, vasculature, or edema of the synapses [41]. While the shrinkage was relatively small (i.e., <4.0%) for the brain sections overall, correcting for processing-induced shrinkage (or swelling) is important for increasing the accuracy of the data [31]. Hence, a shrinkage correction was applied in the current study to ensure that any statistically significant structural changes between the repeated hypoxic and the repeated normoxic rats reflected the real extent of the difference. The real section thickness of the serial ultrathin sections was also measured and used for the volume calculations, as described in Section 4.

### 2.5. The Repeated Hypoxic Rats Exhibited Hyperactive Behaviour in the Staircase Test

The behaviour of the rats was assessed by measuring the number of entries onto a platform in the staircase test. Across PN141–150, the repeated hypoxic rats made a statistically significant higher number of entries onto the platform than the repeated normoxic rats (Figure 7c). Post hoc analysis revealed a specific increase on PN150. Hence, the repeated hypoxic rats were significantly more hyperactive compared to the repeated normoxic rats. These results are consistent with the findings of previous studies, which showed that repeated hypoxia during the equivalent of extreme prematurity induces hyperactivity, specifically ADHD-like hyperactivity in response to delayed reward ([24,25]; see also Section 3). The duration of time spent on the platform per entry revealed no statistically significant differences between the two groups of rats across time (two-way ANOVA, interaction *p* = 0.77, time alone *p* = 0.40, treatment alone, *p* = 0.75).

### 2.6. Comparison of Results for an ADHD Risk Factor Versus a Schizophrenia Risk Factor

**Figure 8:**
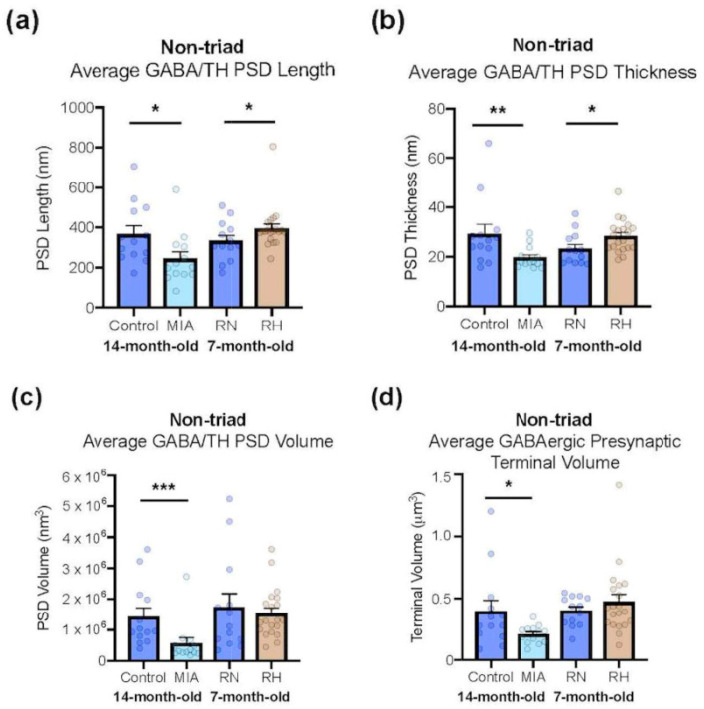
Quantitative differences in the ultrastructure of non-triadic synapses within the pVTA of MIA rats, saline-injected control rats, repeated hypoxic rats and repeated normoxic control rats. In non-triadic synapses formed between RMTg GABAergic axon terminals and pVTA dopaminergic dendrites (i.e., GABA/tyrosine hydroxylase [TH] synapses), MIA rats have a significantly *shorter* (**a**), *thinner* (**b**) and *volumetrically smaller* (**c**) postsynaptic density, as well as a significantly *smaller* RMTg GABAergic presynaptic terminal volume (**d**), compared to the control rats. At the same type of synapses, the repeated hypoxic rats have a significantly *longer* (**a**) and *thicker* (**b**) postsynaptic density compared to the repeated normoxic controls. * *p* < 0.05, ** *p* < 0.01, *** *p* < 0.001. Original data for the MIA rats are from Seo et al. [31], with copyright approval. MIA, maternal immune activation; RN, repeated normoxic; RH, repeated hypoxic.

Figure 8 illustrates the data from the current study compared to the effect of a schizophrenia risk factor in Seo et al. [31]. Seo et al. [31] used the same double immunohistochemical and stereological methods as in the current paper. Strikingly, synaptic measurements in the pVTA GABA-TH non-triads generally show an increase in the repeated hypoxic ADHD-like condition, and a decrease in response to a schizophrenia risk factor, compared to the control conditions. There are also specific statistically significant increases in response to repeated hypoxia, and statistically significant decreases in response to a schizophrenia risk factor, compared to their respective control group (Figure 8). These findings are consistent with the original hypothesis of opposite effects in the same synapses. The specific direction of the effects for the repeated hypoxia and MIA rats is also consistent with this hypothesis (see also Section 3). Notably, in addition, the average values for the two control groups are very similar, indicating the reproducibility of the results across control conditions and adult ages.

**Figure 9:**
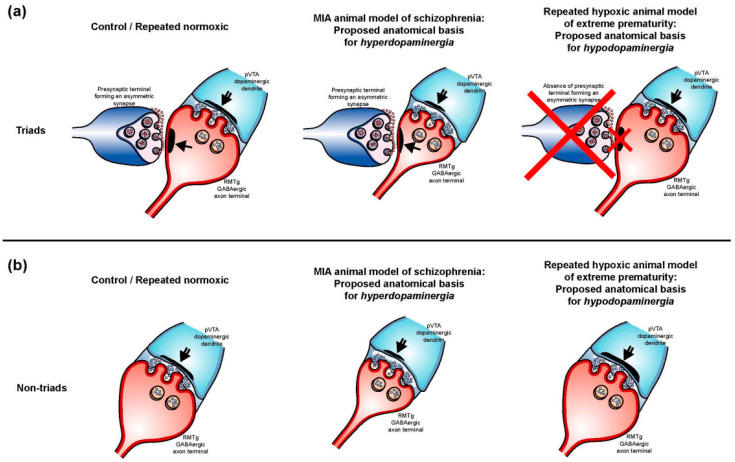
Illustrations of the opposite changes in MIA versus repeated hypoxic rats in the pVTA. Graphical illustrations of synaptic *triads* (**a**) and *non-triads* (**b**) in the pVTA of control/repeated normoxic, MIA or repeated hypoxic rats. In *synaptic triads*, MIA rats have significantly *shorter* and *volumetrically smaller* PSDs at RMTg GABAergic/pVTA dopaminergic synapses. MIA rats also have a significantly *smaller volume* of RMTg GABAergic axon terminals, as well as *thinner* PSDs for asymmetric synapses, compared to the controls. The repeated hypoxic rats *lack* asymmetric synaptic input onto the RMTg GABAergic axon terminals that synapse with pVTA dopaminergic dendrites. For synaptic non-triads, MIA rats have a *decreased* length, thickness and volume of PSDs at RMTg GABAergic/pVTA dopaminergic synapses, as well as a *reduced* volume of the RMTg GABAergic axon terminals, compared to the controls. In contrast, the repeated hypoxic rats have *increased* length and thickness of PSDs at RMTg GABAergic/pVTA dopaminergic synapses compared to the repeated normoxic controls. The double arrow indicates a symmetric synapse formed between a RMTg GABAergic axon terminal and a pVTA dopaminergic dendrite. The single arrow indicates an asymmetric synapse formed between a presumed glutamatergic axon terminal and a RMTg GABAergic axon terminal. Original data for the MIA rats are from Seo et al. [31], with copyright approval. MIA, maternal immune activation.

The statistical differences observed in the non-triadic synapses between repeated hypoxic and repeated normoxic rats, and between the MIA rats and their controls, are illustrated schematically in Figure 9. The absence of synaptic triads in repeated hypoxic rats is also illustrated in Figure 9.

## 3. Discussion

Hypofunctioning in the dopamine pathway from the midbrain VTA to the forebrain is implicated in ADHD [5,20]. Since ADHD is a neurodevelopmental disorder [2], a chronic condition [21], and long-term changes in nervous system function are associated and/or maintained by changes in synaptic structure [22], we investigated if there are structural synaptic changes in the midbrain VTA in adult rats exposed to an ADHD risk factor during development. Currently, technology restricts this type of study to animals. We detected long-term anatomical alterations in GABAergic synaptic inputs on dopaminergic neurons in the midbrain pVTA that likely relate to a characteristic feature of ADHD: decreased striatal dopamine release contributing to hypodopaminergia. The results provide evidence of a specific site that could be targeted with new drug therapies to treat ADHD, particularly the hyperactive/impulsive presentation/subtype.

### 3.1. Detailed Synaptic Findings and Therapeutic Implications

For the specific findings, we demonstrated long-term structural changes, at 7 months- of-age, in RMTg GABAergic synaptic inputs onto pVTA dopaminergic neurons in a repeated hypoxic animal model of the hyperactive/impulsive presentation of ADHD. Exposure to repeated hypoxia or repeated normoxia occurred at PN1–3. Our first major finding was that repeated hypoxic rats had a significantly thicker (22%) and longer (18%) PSD at RMTg GABAergic-pVTA dopaminergic synapses when compared to control repeated normoxic rats. These long-term anatomical alterations could underlie the enhanced GABAergic inhibition of the pVTA dopaminergic neurons, leading to decreased dopamine release in the forebrain. Larger active zones and PSDs at GABAergic synapses are correlated with increased release probability and efficacy of inhibition [42,43]. It is acknowledged that an alternative possibility is that the changes observed may not yield hypofunction, and, instead, perhaps only a change in how inhibition is modulated.

These long-term anatomical alterations may reflect an increase in the number of postsynaptic receptors and/or scaffolding proteins in the PSD of the RMTg GABAergic synapses on dopaminergic neurons in the pVTA. In this context, the anatomical findings could represent a step towards novel targeted pharmacological approaches that use specific antagonists for receptors, and small-molecule and peptide modulators of scaffolding proteins in the PSD, at the identified synapses in the pVTA [44,45]. To facilitate these approaches, increased knowledge is required of the type of GABA receptors and their subunits, and the peptide modulators in the PSD, at the identified synapses. For example, it is known that the VTA dopaminergic neurons express mRNA for six GABA_A_ receptor units (α2, α3, α4, β1, β3, γ2, [46]), which mediate fast inhibitory neurotransmission. Which specific mRNAs are translated into protein at the identified synapses is currently unknown. This is the next level of complexity that should be considered to understand the ultrastructural findings reported here and to advance novel targeted pharmacological approaches.

While the repeated hypoxic rats had a significantly thicker and longer PSD at RMTg GABAergic-pVTA dopaminergic synapses, we did not find a statistically significant difference in the volume of PSDs between the two groups. The length was the maximum length measured of a PSD, and the width was the maximum width measured, across the different serial sections that contained each specific synapse. No change in volume could be partially attributed to the wide variation in the total three-dimensional span (and hence volume) of the PSDs, which ranged between 2 and 15 ultrathin sections for both repeated normoxic and repeated hypoxic rats (Figure 6c,g). The lack of change in volume may also be due to an increased complexity of PSDs, yielding more gaps, in the repeated hypoxic rats. An increased sample size is required to investigate the potential change in the shape of PSDs. The statistically significant structural changes observed at the PSD are still likely to contribute to functional changes because the area of the PSD at GABAergic synapses is correlated with increased release probability and efficacy of inhibition [42,43]. If the increased thickness and length of the PSD represents an increased number of GABAergic receptors, then previous research indicates that the distribution of quantal amplitudes parallels that of synaptic GABA_A_ receptor number [47]. 

Repeated hypoxic rats also have a statistically significant loss of dopaminergic neurons in the VTA, but not in other midbrain dopaminergic regions [24]. In combination with the synaptic findings of the current study, there are now two anatomical results, at the cellular and synaptic level, respectively, that could contribute to hypodopaminergia and ADHD-like hyperactivity and impulsivity in repeated hypoxic rats. Both findings contribute to further elucidation [4] of the underlying neuropathology of a specific presentation/subtype of ADHD.

### 3.2. Presence or Absence of Synaptic Triads

A second major finding was the confirmation of previous results [31] of the presence of axoaxodendritic synaptic triads in the pVTA. This finding was confirmed in the current study in a different set of control (i.e., normal) rats compared to Seo et al. [31]. We also extended the previous findings to include animals of ~7 months-of-age. The synaptic triads were previously observed in the pVTA at 3 and 14 months-of-age [31]. The synaptic triads involve a putative glutamatergic axon terminal synapsing onto a RMTg GABAergic axon terminal, which in turn synapses onto a pVTA dopaminergic dendrite [31]. Together, these results indicate that the synaptic triads are a consistent component of the synaptic circuitry of the normal rat pVTA throughout early to middle adulthood. Triads involving axoaxodendritic synapses have also been observed in other regions of the central nervous system, including the rat trigeminal nucleus [48], the cat cerebellum [49] and the monkey thalamus [50].

A third major finding was the lack of detection of synaptic triads in the repeated hypoxic rats. This seems somewhat paradoxical, as the absence of a putative glutamatergic boost onto RMTg GABAergic axon terminals would be expected to lessen an enhanced inhibition of pVTA dopaminergic dendrites by RMTg GABAergic axon terminals in the repeated hypoxic rats. There is the possibility that the absence of the putative glutamatergic boost, perhaps due to a developmental deficit in inputting glutamatergic axons, leads to a compensatory restructuring mechanism at the synapse (i.e., increased thickness of the PSD) in the repeated hypoxic brain, which in turn leads to hypodopaminergia. There is substantial experimental evidence of activity-driven synaptic plasticity at GABAergic synapses [42]. To explore this hypothesis, the synaptic circuitry of younger repeated hypoxic rats could be investigated for the presence of synaptic triads. However, the methods used in the current study could not be applied because the rats need to be at least 6 weeks old for accurate injection of the LV into the RMTg, and then at least 6 weeks is required for the GFP marker protein to be fully expressed in the RMTg following LV vector transduction [31]. Hence, alternative methods for labelling specific neuronal populations would be required in younger animals.

The lack of detection of synaptic triads in the pVTA of repeated hypoxic rats may contribute to the loss of a mechanism that filters signals the animal should not do, contributing to ADHD-like impulsivity. Specifically, a lack of glutamatergic inputs onto the GABA presynaptic terminals in the repeated hypoxic rats could lead to the omission of critical timed glutamatergic input for accentuating what the animal should respond to and what to not worry about. In this context, all behaviours or outcomes may appear to be equally important because too much neural processing is occurring. These hypotheses require further study. Whether these anatomical findings, and hypothesized functional consequences, are relevant to impulsivity in human ADHD remains to be investigated.

The lack of detection of synaptic triads in the repeated hypoxic rats is in marked contrast to rats exposed to a schizophrenic risk factor, which showed a statistically significant increase in the number of synaptic triads per section of pVTA compared to control rats [31]. Hence, the repeated hypoxic rats exhibited less complex synaptic triad circuitry in the pVTA compared to normal rats and to rats exposed to a schizophrenic risk factor. Future studies are required to link the absence, presence, or increased presence of structural complexity with specific functional outcomes for these pVTA synaptic triads.

### 3.3. Some Synaptic Changes in the Midbrain of ADHD-like Hyperactive Rats Are Opposite to Those Observed in MIA Rats

A fourth major finding (Figure 8 and Figure 9) was that some effects on the internal synaptic structure in the current study were opposite to that observed in MIA rats that model a schizophrenia risk factor [31]. Specifically, for the synaptic triads, the PSD of the putative glutamatergic synapses, which innervated RMTg GABAergic axon terminals, was significantly *thinner* in the MIA rats compared to the control rats. Furthermore, for both triads and non-triads, the length and thickness of the PSD of RMTg GABAergic-pVTA dopaminergic synapses were significantly *decreased* in the MIA rats compared to the controls. The non-triads of MIA rats also had a significantly *decreased* PSD length, thickness and volume at RMTg GABAergic-pVTA dopaminergic synapses. These specific changes are opposite to the findings in the repeated hypoxic rats, where they had an *increased* PSD thickness and length at non-triadic RMTg GABAergic-pVTA dopaminergic synapses compared to the controls. Such generally opposite anatomical changes seem likely to contribute to opposing dopaminergic conditions, specifically hyperdopaminergia in schizophrenia and hypodopaminergia in ADHD.

The generally contrasting anatomical results are also consistent with clinical observations that inattention, rather than attention, is a prominent shared symptom in co-morbid ADHD and schizophrenia [51]. Inattention is not evident in the repeated hypoxic animal model used, only ADHD-like hyperactivity and impulsivity [24,25,26]. Collectively, these findings suggest that the inattentive presentation of ADHD may have different changes in the brain compared to the hyperactive–impulsive presentation of ADHD and schizophrenia. It remains to be determined whether the changes in the pVTA synaptic connectivity reported in the current study are unique to the hyperactive–impulsive presentation of ADHD. The answer to this question would represent a marked step towards understanding the underlying causal mechanisms of the different presentations/subtypes of ADHD, which are yet to be clearly delineated [4].

Overall, the observed data are consistent with the hypotheses of this study, indicating that future corroboration in additional animals, for the repeated normoxic and repeated hypoxic groups, is merited (see also Potential Limitations below).

### 3.4. Staircase Test as a Measure of Hyperactivity

The behaviour of the rats was assessed by measuring the number of entries on a platform during the staircase test. The repeated hypoxic rats made a statistically significant higher number of entries compared to the repeated normoxic rats. While the staircase test primarily measures skilled forepaw ability, Söderlund et al. [52] have reported that significantly more spontaneously hypertensive rats, another animal model of ADHD [53], exhibited active exploring behaviour compared to control rats. Specifically, the spontaneously hypertensive rats had significantly more voluntary venturing onto the staircase platform to access the sugar pellets. Their findings are similar to the current study. Hence, the number of entries made onto the platform in the staircase test provides an indication of the level of activity in at least two animal models of ADHD. Katzav et al. [54] used another version of the staircase test and recorded the number of stair-climbing events as a measure of hyperactivity in mice, and the number of rearing events as exploration. Their use of a staircase test as an indicator of hyperactivity is consistent with the strategy used in the current study and by Söderlund et al. [52]. The hyperactivity observed in the current study could be relevant to the changes in synaptic connectivity and structure that were detected in the same rats.

In previous studies, ADHD-like hyperactivity has been detected in 10–11 repeated hypoxic rats, compared to 8 repeated normoxic rats, on a standard, ADHD-specific delayed reward, fixed interval test at 3 and at 16 months-of-age [24,25]. Thus, ADHD-like hyperactivity has been consistently detected in this animal model.

### 3.5. Potential Limitations

Extensive behavioural testing in the current study, including food restriction, could have influenced the anatomical findings. To avoid this caveat, a parallel behavioural-only cohort, and a parallel anatomical-only cohort, could be included in future studies to confirm consistency in the behavioural and anatomical findings across three cohorts at 7 months-of-age.

The statistical approach used in this study for the synaptic data from the repeated normoxia and repeated hypoxia-ADHD-like groups is based on synapse-level analysis. While this is a common procedure in this type of study (e.g., [34]), it has a potential limitation in that the results may not be representative of effects across animals. For the current data, Figure 6e–h,j show that no one animal carried the findings for either group. It is also noteworthy that the average synaptic data from the two different control groups (involving two rats per group, *n* = 4) are very similar for the comparative results for the ADHD cohort and the schizophrenic cohort (Figure 9). This indicates the reproducibility of the results across control conditions and adult ages. This provides additional evidence that the results are likely to be representative.

### 3.6. Males Versus Females

We used male rats in this study because ADHD is more common in human males with a male to female ratio of about 3:1 for all three subtypes (i.e., presentations, [27]). Female humans also present differently, with the inattentive subtype being more prevalent [27]. In children born extremely prematurely, ADHD is the most common neurobehavioural disorder (see Section 1), and males are more at risk [55]. Male rats exposed to repeated hypoxia during the equivalent of extreme prematurity exhibit features that resemble the hyperactive–impulsive subtype [24]. The incidence of schizophrenia is also higher among men than women, with a ratio of nearly 1.4:1 [56], and the results from Seo et al. [31] are from male rats. For all these reasons, our results in this paper are likely to be specifically relevant to males.

In males, the long-term effect of neonatal hypoxia on the brain may involve testosterone. It is well documented that a neonatal surge in plasma testosterone occurs shortly after birth in both male rats and humans [57]. This increase in testosterone at a critical period of development has been implicated in the long-term modulation of a number of brain functions, including dopaminergic neurotransmission [58]. The neonatal testosterone surge is relatively labile in that it has been shown to be altered by environmental manipulations such as maternal exposure to repeated hypoxia [59] and birth hypoxia at PN0 in male rats [60]. Hence, there is the possibility that the repeated hypoxia used in the current study, over PN1–3 in male rats, influences testosterone levels, which in turn affects long-term dopaminergic neurotransmission. This specific hypothesis requires investigation.

In male versus female rats, an hypoxic insult at PN0 has differential long-term effects on brain monoaminergic systems [61]. Specifically, birth hypoxia at PN0 may dysregulate neonatal testosterone secretion in male pups, resulting in long-term effects on cerebral dopamine levels in males but not in females [61]. In females, lifetime exposure to estrogen appears to increase responsiveness to amphetamine [58], which could be advantageous in the treatment of ADHD. During the development of the nigrostriatal dopaminergic neurons, the action of estrogen includes the induction of the dopaminergic phenotype, the maturation of dopamine neuronal characteristics in a functional and morphological way, the stabilization of neural connectivity and synapses and the survival of this cell population during development, but also under neurotoxic conditions [62]. Whether the same effects occur for VTA dopaminergic neurons appears unknown. Overall, being female with estrogen appears to confer protection from the effects of neonatal hypoxia on midbrain dopamine neurons.

### 3.7. More General Comments: Anti-Psychotics, Exercise and Gene Expression

The findings in the current study, and in a previous study on MIA related to schizophrenia [31], were detected after either repeated hypoxia or MIA in rats during the equivalent of the human prenatal period (i.e., during the second trimester in humans). Thus, the effects of hypoxia during later life are not specifically related. However, it should be noted that different generations of antipsychotics for the treatment of schizophrenia have differential effects on regional cerebral blood flow, thereby decreasing or increasing oxygenation [63]. In addition, exercise has been shown in multiple findings to be a useful treatment for improving positive and negative symptoms and cognitive functioning in schizophrenia [64]. Aerobic exercise may specifically reduce the physical health problems associated with schizophrenia and offset antipsychotic side effects, such as metabolic syndrome [64]. 

In studies on gene expression, it has been reported that intermittent hypoxia elicits reduced tyrosine hydroxylase recruitment and phosphorylation compared with sustained hypoxia [65]. However, this is in adult rats. There are currently no gene expression studies on the neonatal repeated hypoxic animal model used in this study. Thus, further research is needed to determine whether changes in gene expression contribute to the mechanisms underlying the observed synaptic anatomical changes.

## 4. Materials and Methods

### 4.1. Animals and Overall Experimental Procedures

The experimental timeline of this study is shown in Figure 1c. All experimental procedures were approved by the Animal Ethics Committee of the University of Otago. The dams, pups and weaned rats were housed in a temperature- and humidity-controlled room that was maintained on a 12 h light/dark cycle. Food and water were available ad libitum, except prior to and during behavioural experiments, when the animals were placed on a restricted diet. The animals were weighed daily from postnatal day (PN) 0 to PN17 and at least twice weekly thereafter to monitor their wellbeing over the course of this study.

### 4.2. Repeated Hypoxic Model of ADHD-like Hyperactivity and Impulsivity

Male Sprague Dawley rat pups from two litters were used. The day of their birth was assigned as PN0. At PN1–3 (Figure 1c), the pups were exposed to repeated bouts of either hypoxia or normoxia as described previously [24,25,26]. In brief, the repeated hypoxic pups were exposed to humidified 1.5% oxygen, 5% carbon dioxide and 93.5% nitrogen at 37 °C every 2 h. Since the brain is more vulnerable to a set level of hypoxia (e.g., 8%) at older ages and much less vulnerable to the same concentration during early development, we used 1.5% oxygen in this study on PN1–3 rats. Each hypoxic exposure lasted 15 min on PN1 and 14 min on PN2 and PN3. The maximum survivable insult was 15 min on PN1 and 14 min on PN2 and PN3, as determined from pilot experiments [25]. The first exposure commenced at 7:00 A.M. and the last exposure commenced at 7:00 P.M. This yielded a total of seven exposures a day over 12.25 h. Repeated normoxic pups underwent the same experimental paradigm, except that the exposure was to humidified normal air at 21% oxygen with a balance of nitrogen. At the end of each exposure, all pups were returned to their respective dam for feeding and recovery. Some of the repeated hypoxic or repeated normoxic pups from the two litters were used in other experiments not reported here.

### 4.3. LV Vector Injection into the RMTg

An LV vector containing a promoter for glutamate decarboxylase-67 (GAD-67) enabled the transduction and visualization of rat RMTg GABAergic neurons in vivo. An LV construct, LV-GAD67-GFP (Systems Biosciences https://www.systembio.com/products/imaging-and-reporter-vectors/stem-cell-reporters/neural/mouse-gad67-pgreenzeo-differentiation-reporter, re-accessed on 15 June 2023), which expresses copGFP (green fluorescent protein) under the control of the mouse GAD-67 promoter, was packaged into second-generation LV vectors and pseudotyped using a vesicular stomatitis virus glycoprotein envelope by the Otago Viral Vector Facility [66] and the Brain Research New Zealand Mārama Viral Vector Platform (www.brnz.ac.nz, accessed on 26 October 2016). The packaging and use of this vector were approved by the New Zealand Environmental Protection Agency (approval #GMD002854). The viral genomic titre was 3.95 × 10^10^ viral genomes/mL. After injection into the RMTg, selective expression of this viral vector with a GAD-67 promoter has been previously established in our laboratory (see Figure 2 in Seo et al. [31]). Specifically, GFP colocalized within GAD-67-positive somata in the RMTg.

At PN48 (i.e., from ~2 months-of-age, Figure 1c), two male repeated hypoxic rats (238 g, 236 g) and two control male repeated normoxic rats (279 g, 284 g), one of each from two different litters, were injected with the LV vector LV-GAD67-GFP bilaterally. Each rat was anesthetized (ketamine/domitor/atropine, 75/0.5/0.06 mg/kg s.c.) and mounted in a stereotaxic frame, while its body temperature was maintained at 37 °C using heating pads. For each cerebral hemisphere, a hole was drilled through the skull at anteroposterior (AP) -6.7 mm, mediolateral (ML) 1.5 mm, relative to the bregma [67]. The LV vector (100 nL per hemisphere) was pressure-injected into the RMTg, at dorsoventral (DV) 8 mm relative to the dura, at a rate of 50 nL/min using a 10 µL Hamilton syringe and a UMP-3 pump (World Precision Instruments, Sarasota, FL, USA). Amphoprim (30 mg/kg s.c.) and carprofen (5 mg/kg s.c.) were administered prophylactically. Antisedan (2.5 mg/kg s.c.) was given as a reversal agent for the anesthesia. The animals were then monitored twice daily for 4 days post-operation to ensure their welfare.

### 4.4. Behavioural Testing

The repeated hypoxic and repeated normoxic rats, which had been injected with the LV vector (n = 4), were also behaviorally tested using a modified version of the staircase test [26,68] from PN132–150 (i.e., from ~4 months-of-age, Figure 1c, 5 days per week). Since it takes at least 6 weeks for the GFP marker protein to be fully expressed in the RMTg following LV vector transduction [31], testing from ~4 months-of-age ensured that the behavioural testing did not affect the full expression of the GFP marker protein. To motivate the rats to complete the test, they were food-restricted from PN127 for 5 days to 85% of their free-feeding weight. They were kept at this weight until the conclusion of the behavioural study. While the staircase test primarily measures skilled forepaw ability (as detailed below), it was used here to record and analyze the number and duration of entries made by a rat onto the platform during PN141–150 to assess its hyperactivity (see below and Section 3).

In brief, one staircase (right or left side) was baited per trial in a random order. Each rat was placed inside the entry chamber of the staircase testing apparatus. The entry chamber provided access to a platform chamber (https://lafayetteneuroscience.com/products/rat-staircase, re-accessed on 30 April 2023). Adjacent to the platform were sucrose pellets on the right or left staircase (one pellet each on the top two steps and three pellets each on the bottom five steps), as well as a small piece of chow placed on each step as an olfactory cue. In the first week, the rats were trained for five consecutive days, 15 min per side, to enter the platform and reach out, grasp and eat the pellets. In the second and third week, the rats were allowed 5 min per side and the number of entries onto the platform, and the time spent on the platform per entry, was recorded (see below).

The rat can leave the platform chamber at any stage during the testing and thus re-enter the entry chamber. This can occur when a rat, for example, has difficulty grasping and eating the sugar pellets from a staircase located alongside the platform, or it drops a sugar pellet further down a staircase (i.e., it is not receiving a food reward). The rat returns to the platform chamber because it knows the food reward is located on the staircase next to the platform. This response was used as a measure of hyperactivity, specifically the number of entries onto the platform and the time spent on the platform per entry. The number of sugar pellets eaten, and the height of the step from which the pellet was obtained, are both considered measures of skilled forepaw ability, specifically grasping or reaching [69]. Hence, they were not used as measures of hyperactivity in this study.

It is acknowledged that the gold-standard method for assessing ADHD-like hyperactivity, in response to delayed reward, is a fixed interval-extension paradigm in operant chambers [24,25,26,53,70]. In previous studies, ADHD-like hyperactivity has been detected in repeated hypoxic rats using this gold-standard test [24,25]. The repeated hypoxic rats in these previous studies were exposed to the same animal model as the current study. From PN154 to PN196 (over 43 days, when 5–6.5 months old), the rats in the current study were habituated, trained and tested on this fixed interval-extinction test (as in Oorschot et al. [25]; Kohe et al. [24]). This test required habituation to an operant chamber (2 days), then training to access a food hopper (1 day minimum) and then training to press a lever to obtain food and reach the criterion in this performance (minimum of 8 days; see Table 1 in Oorschot et al. [25]). This is followed by testing for 32 days [25]. For the cohort of four rats in the current study, only one rat learned to press a lever to obtain a food reward and completed the testing after a total duration of 43 days. The other three other rats achieved food hopper training in 2 days but did not achieve the criterion for lever press training over the subsequent 41 days. We persevered in the hope that they could be trained, but they could not be progressed to the fixed interval-extension testing phase. Hence, the staircase test was used as an alternative method to measure the rats’ level of activity (see also Section 3).

### 4.5. Double Immunohistochemical Staining

The two repeated hypoxic and the two repeated normoxic rats that had been injected with the LV vector and behaviorally tested were euthanized between PN203–231 (average age 7.3 months, 552–625 g, Figure 1c). The variation in the timing of the perfusion was due to logistical constraints, specifically, the required processing of serial double immunostained 50 µm vibratome sections (4 days per rat) for subsequent transmission electron microscopic analyses. The completion of this processing immediately after each perfusion was necessary for the optimum visualization of the synapses.

Each rat was anesthetized with ketamine/xylazine (139/13.9 mg/kg i.p., respectively) and intracardially perfuse-fixed with 2% paraformaldehyde and 0.1% glutaraldehyde in phosphate buffer (PB; 0.1 M, pH 7.2). Each brain was dissected and post-fixed overnight at 4 °C in the fixative. On the following day, the left and right cerebral hemispheres were serially sectioned in the sagittal plane (at an angle of 7° [31]) at a thickness of 50 µm on a vibratome (Vibratome 1500, Leica Biosystems, Nussloch, Germany). Sections containing the RMTg were examined using an Olympus AX70 fluorescent microscope to confirm GFP expression in the region (Figure 2). Sections containing the pVTA were processed further for immunohistochemistry and electron microscopy (EM).

Antibodies to copGFP and tyrosine hydroxylase, coupled with different end-labels, were used, respectively, to label virally transduced RMTg GABAergic neurons and pVTA dopaminergic neurons for electron microscopic visualization (Figure 1d,e).

All immunohistochemical and EM processing steps were undertaken at room temperature (RT), unless otherwise specified. Free-floating 50 µm brain sections were washed in phosphate-buffered saline (PBS; 0.1 M, pH 7.4) (3 × 5 min), incubated with 50% ethanol (30 min) to improve antibody penetration [71], and then washed in PBS again (3 × 5 min). The sections were blocked with 5% normal goat serum (NGS) (Hercus Taieri Resource Unit) in PBS for 1 h to minimize non-specific binding prior to overnight incubation at 4 °C in a cocktail of two primary antibodies in diluting buffer (1% BSA, Sigma, St. Louis, MO, USA; in PBS). Anti-turbo GFP (tGFP) monoclonal mouse clone 2H8, horseradish peroxidase-conjugated primary antibody (OriGene, Rockville, MD, USA, TA150043, 1:100, Table 1) was used to label virally transduced GABAergic neurons in the RMTg. Polyclonal affinity purified rabbit anti-TH primary antibody (Millipore, Burlington, MA, USA, AB152, 1:5000, Table 1) was used to label dopaminergic neurons in the pVTA. The characterization of these antibodies has been previously described [31].

**Table 1:** Primary antibodies.

Target Protein	Antigen	Species, RRID	Dilution	Source
Tyrosine hydroxylase	Purified from rat pheochromocytoma	Rabbit polyclonal IgG, RRID:AB_39024	1:5000	Millipore, catalogue No. AB152
Glutamic acid decarboxylase 67 kDa isoform (GAD-67)	Recombinant GAD67 protein	Mouse monoclonal IgG, RRID: AB_2278725	1:500	Millipore, catalogue No. MAB5406

The sections were washed with diluting buffer (3 × 5 min) before being incubated overnight at 4 °C in ultrananogold-conjugated goat anti-rabbit secondary antibody (Aurion, Wageningen, The Netherlands, 25360, 1:50) in diluting buffer with 5% NGS. The sections were washed in PBS (3 × 5 min) and incubated for 10 min in 0.05% 3,3′-diaminobenzidine (DAB), 0.003% H_2_O_2_ and 0.03% nickel chloride to yield an electron-dense black reaction product to visualize virally transduced RMTg GABAergic neurons at the electron-microscopic level. The sections were washed in PBS (2 × 3 min) to stop the DAB reaction, then further washed in PBS (3 × 5 min) and PB (2 × 10 min). The sections were post-fixed for 15 min in 2% glutaraldehyde and washed in PB (2 × 10 min) and conditioning solution (Aurion, Wageningen, The Netherlands, 25830) (4 × 10 min), before being incubated in silver enhancement mixture (Aurion, Wageningen, The Netherlands, 25521) for 90 min. This step was necessary to silver-enhance the ultrananogold labels in dopaminergic neurons in the pVTA, effectively yielding two unambiguous end-labels for electron microscopy (DAB and silver-enhanced ultrananogold) [31]. The sections were washed in conditioning solution (4 × 10 min) and PB (2 × 10 min) before being incubated in 0.5% osmium tetroxide for 30 min.

After washing in PB (3 × 5 min), the sections underwent a series of dehydration steps as follows: 50% ethanol (5 min), 70% ethanol (5 min), 95% ethanol (5 min), 100% ethanol (3 × 5 min) and propylene oxide (PO; 2 × 5 min). The sections were then infiltrated with resin through the following steps: 1:3 epoxy EMbed812 resin to PO (30 min), 1:1 resin to PO (30 min), 3:1 resin to PO (30 min), resin (1 h), resin (overnight) and resin (1 h). The sections were flat-embedded in resin between silanized slides and cured for 24 h at 60 °C. One flat-embedded 50 µm section from each rat containing TH-positive dopaminergic neurons in the pVTA and GABAergic-positive axons coursing nearby was serially cut at a thickness of 80 nm using a Leica EM UC7 Ultramicrotome (Leica Microsystems, Wetzlar, Germany). The ultrathin sections were placed on formvar-coated copper slot grids and stained in an ultrastainer (LKB, Mt Waverley, Australia) with 1% uranyl acetate in ddH_2_O for 20 min at 25 °C followed by 3% lead citrate in ddH_2_O for 3 min at 25 °C.

### 4.6. Ultrastructural Investigation of Synaptic Circuitry

**Figure 10:**
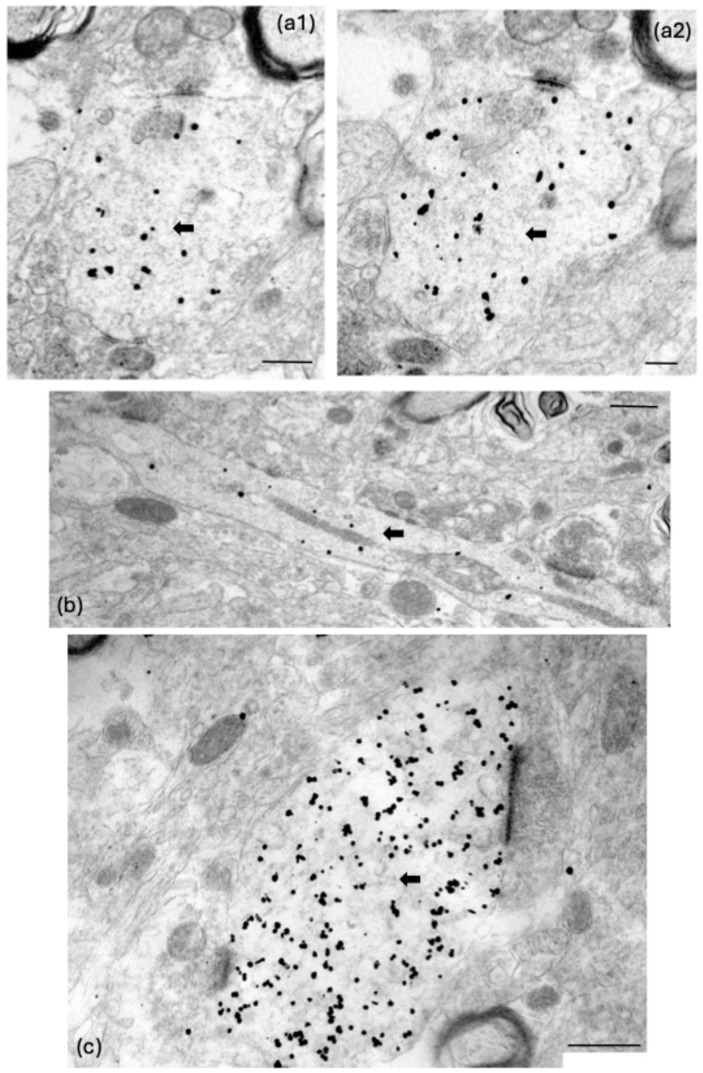
Further evidence of extensive and specific localisation of immunogold particles in dopaminergic postsynaptic dendrites (black arrows) in the rat pVTA. (**a1**,**a2**) An immunolabelled dendrite is imaged in adjacent serial sections (38,39) from a repeated normoxic rat. A different immunolabelled dendrite is illustrated in a repeated hypoxic (**b**), or a repeated normoxic (**c**), rat. Scale bars are as follows: (**a1**,**a2**) 380 nm; (**b**) 240 nm; (**c**) 500 nm.

The sections were viewed using a CM100 transmission electron microscope (Philips/FEI Corporation) and photographed using iTEM imaging platform software (https://cfim.ku.dk/equipment/electron_microscopy/cm100/iTEM_Main_Brochure.pdf accessed on 1 August 2018) via a MegaView II digital imaging camera (Olympus Soft Imaging Solutions, Münster, Germany). The sections were initially scanned at 3400× or 5800× magnification to identify RMTg GABAergic axons (dark, diffuse peroxidase stain) and dopaminergic dendrites (round, black ultrananogold particles; Figure 3, Figure 4, Figure 5 and Figure 10) in the pVTA. When two profiles were located in close proximity to each other, they were examined through multiple serial sections at higher magnifications (between 17,500× and 66,000×) to verify the presence of a synapse formed between them. Synapses made between these neuronal profiles were positively identified if they exhibited a presynaptic density, a postsynaptic density (PSD), a clear synaptic cleft and presynaptic vesicles [31,36,71,72]. 

The postsynaptic dendrites in the pVTA were positively identified as dopaminergic if they exhibited immunogold particles in serial sections through the same cross-sectioned or longitudinally sectioned profile (Figure 3 and Figure 4). Note that only one particle is seen in the dendritic profiles labelled in Figure 3 because a high magnification was necessary to illustrate the synapses. A higher density of gold particles became evident in nearby serial sections (e.g., Figure 4a4). The rare event of a low density of labelling required seeing one or more particles in the same profile in adjacent sections through the serial stack [31]. An absence of gold particles also needed to be consistently exhibited in adjacent structures to indicate specificity (Figure 3 and Figure 4). Extensive, specific immunogold labelling of dopaminergic dendritic profiles was evident in repeated normoxic and repeated hypoxic animals (Figure 4b1,b2,c1–c3 and Figure 10).

Specific features of the identified synapses in the pVTA were quantitatively compared between the repeated hypoxic and the repeated normoxic rats. The specific calibrated magnification used to measure the synaptic features was 17,500× to measure presynaptic GABAergic terminal volume; 33,000×, 46,000× or 66,000× for all measurements of the PSD (length, thickness and area). ImageJ (version 1.51s, National Institutes of Health) was used for all anatomical measurements, with images being digitally magnified in the program to ensure accurate measurement. Brightness and contrast were also optimized in ImageJ to enable clear delineation of membrane boundaries and each PSD. For PSD length, the maximum length within serial sections that contained a synapse’s PSD was measured (Figure 7b) using the segmented line tool. For PSD thickness, the maximum thickness of a PSD within serial sections that contained a synapse’s PSD was measured (Figure 7a,b) using the straight-line tool. For volume measurements of peroxidase-labelled RMTg GABAergic presynaptic terminals, and each PSD, Cavalieri’s method was used ([41,73], Figure 7a,b). This involved using the polygonal selection tool to calculate the area of a presynaptic terminal or PSD in which the terminal or PSD could be clearly identified. This was followed by multiplying the summated area by the average section thickness of each rat to yield the total volume for each structure (Figure 7a,b).

For perforated synapses, the shape of the PSD was defined per synapse. The incidence of each type of shape was then quantified. A simple perforated synapse had a PSD that was continuously evident across the serial sections in the sagittal plane (i.e., there were no gaps in the sagittal plane). There were also one or two perforations present in the coronal plane, and the *same presynaptic terminal present throughout*. A complex perforated synapse had a PSD that was not continuously present throughout the serial sections in the sagittal plane (i.e., there were gaps). There was also one perforation, or two or more perforations, in the coronal plane, and the *same presynaptic terminal present throughout*.

### 4.7. Correction for Section Thickness and Shrinkage

To increase the accuracy of the ultrastructural measurements, the actual section thickness should be measured and used [31]. For each rat, Small’s minimal folds method was used to measure the thickness of the serial ultrathin sections. By this method, section thickness was estimated to be half of the mean width of the measured folds [74,75,76]. The average section thickness were 70.73 nm and 71.02 nm for each repeated normoxic rat, and 71.38 nm and 71.27 nm for each repeated hypoxic rat, which are lower than the nominal setting (80 nm) on the ultramicrotome. These values were used in Cavalieri’s formula to calculate the volume of each measured PSD or RMTg GABAergic presynaptic terminal.

The volume measurements, and the PSD length and thickness measurements, for each rat were also corrected for any processing-induced shrinkage that occurred between vibratome sectioning (i.e., pre-processing) and after resin embedding (i.e., post-processing). Section area was measured using the polygonal selection tool in Image J (version 1.51s, National Institutes of Health) and substituted into the formula [73]: Area shrinkage (p^2^) = area after processing/area before processing. This area shrinkage factor was multiplied by the thickness-adjusted presynaptic terminal or PSD volume measurements of each rat to arrive at the final values for statistical analyses. The average PSD thickness and length measurements were multiplied by the linear shrinkage factor (p), which was determined from the area (p^2^) shrinkage factor [73], to determine the final values for statistical analyses.

### 4.8. Statistical Analyses

For statistical comparisons between the repeated normoxic and repeated hypoxic groups, the data for each measured synaptic parameter were first checked for the normality of the distribution. Since the criteria for normality were not met, Mann–Whitney *U* tests were performed. Each three-dimensionally analyzed synapse (n = 33) was considered as an independent unit ([31,34,36,37]; see also the start of Section 2). Sample sizes were not predetermined with statistical methods, but our sample sizes are similar to those reported in previous publications in the field [31,35,37]. Fisher’s exact test was used to analyze the data on the shape of the PSD. Staircase testing data were analyzed using a two-way repeated measures ANOVA and a Holm–Sidak multiple comparisons post hoc test. All data are expressed as the mean ± S.E.M. (standard error of the mean), and the significance level is indicated as * *p* < 0.05. GraphPad Prism 8 (Graph Pad Software, Inc., Boston, MA, USA) was used for the statistical analyses and the graphing of the data.

## 5. Conclusions

Overall, the anatomical alterations reported in the current paper highlight a particular brain region (i.e., pVTA) and synapses (i.e., RMTg GABAergic-pVTA dopaminergic) that increase our understanding of mechanisms that may underlie dopamine dysregulation in ADHD in males. This core knowledge could guide novel targeted pharmacological approaches that generate therapeutic treatments with less adverse side effects, less potential for abuse, and use in patients in whom current ADHD medications are contraindicated.

## Data Availability

The data presented in this study are available on reasonable request from the corresponding author.
